# Association between genetically proxied glucosamine and risk of cancer and non-neoplastic disease: A Mendelian randomization study

**DOI:** 10.3389/fgene.2024.1293668

**Published:** 2024-06-27

**Authors:** Yingtong Wu, Yinggang Che, Yong Zhang, Yanlu Xiong, Chen Shu, Jun Jiang, Gaozhi Li, Lin Guo, Tianyun Qiao, Shuwen Li, Ou Li, Ning Chang, Xinxin Zhang, Minzhe Zhang, Dan Qiu, Hangtian Xi, Jinggeng Li, Xiangxiang Chen, Mingxiang Ye, Jian Zhang

**Affiliations:** ^1^ Department of Pulmonary Medicine, Xi'an People's Hospital, Xi’an, China; ^2^ First Sanatorium, Air Force Healthcare Center for Special Services, Hangzhou, China; ^3^ Department of Thoracic Surgery, Tangdu Hospital, Air-Force Medical University, Xi’an, China; ^4^ Department of Health Service, Air-Force Medical University, Xi’an, China; ^5^ 94498th Unit of the People’s Liberation Army of China, Nanyang, China; ^6^ Department of Obstetrics and Gynecology, Tangdu Hospital, Air-Force Medical University, Xi’an, China; ^7^ College of Pulmonary and Critical Care Medicine, the 8th Medical Centre of Chinese PLA General Hospital, Beijing, China; ^8^ Department of Respiratory Medicine, Jinling Hospital, Nanjing University School of Medicine, Nanjing, China

**Keywords:** glucosamine, cancer risk, Mendelian randomization, single-nucleotide polymorphisms, causality

## Abstract

**Introduction:**

Observational investigations have examined the impact of glucosamine use on the risk of cancer and non-neoplastic diseases. However, the findings from these studies face limitations arising from confounding variables, reverse causation, and conflicting reports. Consequently, the establishment of a causal relationship between habitual glucosamine consumption and the risk of cancer and non-neoplastic diseases necessitates further investigation.

**Methods:**

For Mendelian randomization (MR) investigation, we opted to employ single-nucleotide polymorphisms (SNPs) as instruments that exhibit robust associations with habitual glucosamine consumption. We obtained the corresponding effect estimates of these SNPs on the risk of cancer and non-neoplastic diseases by extracting summary data for genetic instruments linked to 49 varied cancer types amounting to 378,284 cases and 533,969 controls, as well as 20 non-neoplastic diseases encompassing 292,270 cases and 842,829 controls. Apart from the primary analysis utilizing inverse-variance weighted MR, we conducted two supplementary approaches to account for potential pleiotropy (MR-Egger and weighted median) and assessed their respective MR estimates. Furthermore, the results of the leave-one-out analysis revealed that there were no outlying instruments.

**Results:**

Our results suggest divergence from accepted biological understanding, suggesting that genetically predicted glucosamine utilization may be linked to an increased vulnerability to specific illnesses, as evidenced by increased odds ratios and confidence intervals (95% CI) for diseases, such as malignant neoplasm of the eye and adnexa (2.47 [1.34–4.55]), benign neoplasm of the liver/bile ducts (2.12 [1.32–3.43]), benign neoplasm of the larynx (2.01 [1.36–2.96]), melanoma (1.74 [1.17–2.59]), follicular lymphoma (1.50 [1.06–2.11]), autoimmune thyroiditis (2.47 [1.49–4.08]), and autoimmune hyperthyroidism (1.93 [1.17–3.18]). In contrast to prior observational research, our genetic investigations demonstrate a positive correlation between habitual glucosamine consumption and an elevated risk of sigmoid colon cancer, lung adenocarcinoma, and benign neoplasm of the thyroid gland.

**Conclusion:**

Casting doubt on the purported purely beneficial association between glucosamine ingestion and prevention of neoplastic and non-neoplastic diseases, habitual glucosamine ingestion exhibits dichotomous effects on disease outcomes. Endorsing the habitual consumption of glucosamine as a preventative measure against neoplastic and non-neoplastic diseases cannot be supported.

## Background

Glucosamine, belonging to the category of symptomatic delayed-onset drugs for osteoarthritis (SYSADOAs), represents a form of non-vitamin/non-mineral dietary supplements frequently employed for alleviating joint pain and osteoarthritis (Sherman et al., 2012; Wang et al., 2023). Glucosamine is a widely used supplement among adults in the United States (Conway et al., 2022). The global prevalence of the supplement is noteworthy, with its widespread usage and differential availability across countries. While obtainable without a prescription in Canada, Australia, and the United States, it needs a prescription in several European countries (Sibbritt et al., 2012; Bhimani et al., 2023). Dietary supplements lack the regulatory requirement for US Food and Drug Administration (FDA) approval, distinguishing them from drugs that mandate FDA oversight of both efficacy and safety. Nonetheless, dietary supplements and drugs are often conflated in the public perception. In addition, the effectiveness of glucosamine in alleviating joint discomfort and the symptoms of osteoarthritis has been widely debated (Clegg et al., 2006; Runhaar and van der Wouden, 2010; Wilkens et al., 2010). While dietary supplements are generally deemed safe, they can present a double-edged sword of inherent risks and side effects similar to drugs (Brown, 2017). Consequently, there is a noticeable lack of consensus regarding the benefit of habitual glucosamine consumption in treatment guidelines (American College of Rheumatology Subcommittee on Osteoarthritis Guidelines, 2000; Cutolo et al., 2015; Arden et al., 2021).

The putative anti-inflammatory properties of glucosamine, coupled with its potential utility as a prophylactic agent in the context of malignancy, have been suggested by human and animal studies (Kantor et al., 2012; Ibanez-Sanz et al., 2020; Lee et al., 2020; Kantor et al., 2022; Li et al., 2022; Mazzucchelli et al., 2022; Zhang et al., 2022; Li et al., 2023). Despite the numerous observational studies indicating a protective association between habitual glucosamine consumption and the risk of cancer and non-neoplastic diseases, the potential hazard and causal nature of this connection remain uncertain. This uncertainty stems from the susceptibility of observational studies to confounding factors and reverse causation, which could potentially bias study results. Therefore, it is of paramount clinical significance to elucidate the impact of glucosamine intake in these contexts, enabling informed decision-making in patient care. The fundamental aim of this study was to conduct a meticulous reassessment of the association between genetically predicted habitual glucosamine consumption and the risk of noncommunicable diseases.

## Methods

### Assumptions

All Mendelian randomization (MR) analyses were conducted under the following assumptions (Labrecque and Swanson, 2018).

**FIGURE 1:**
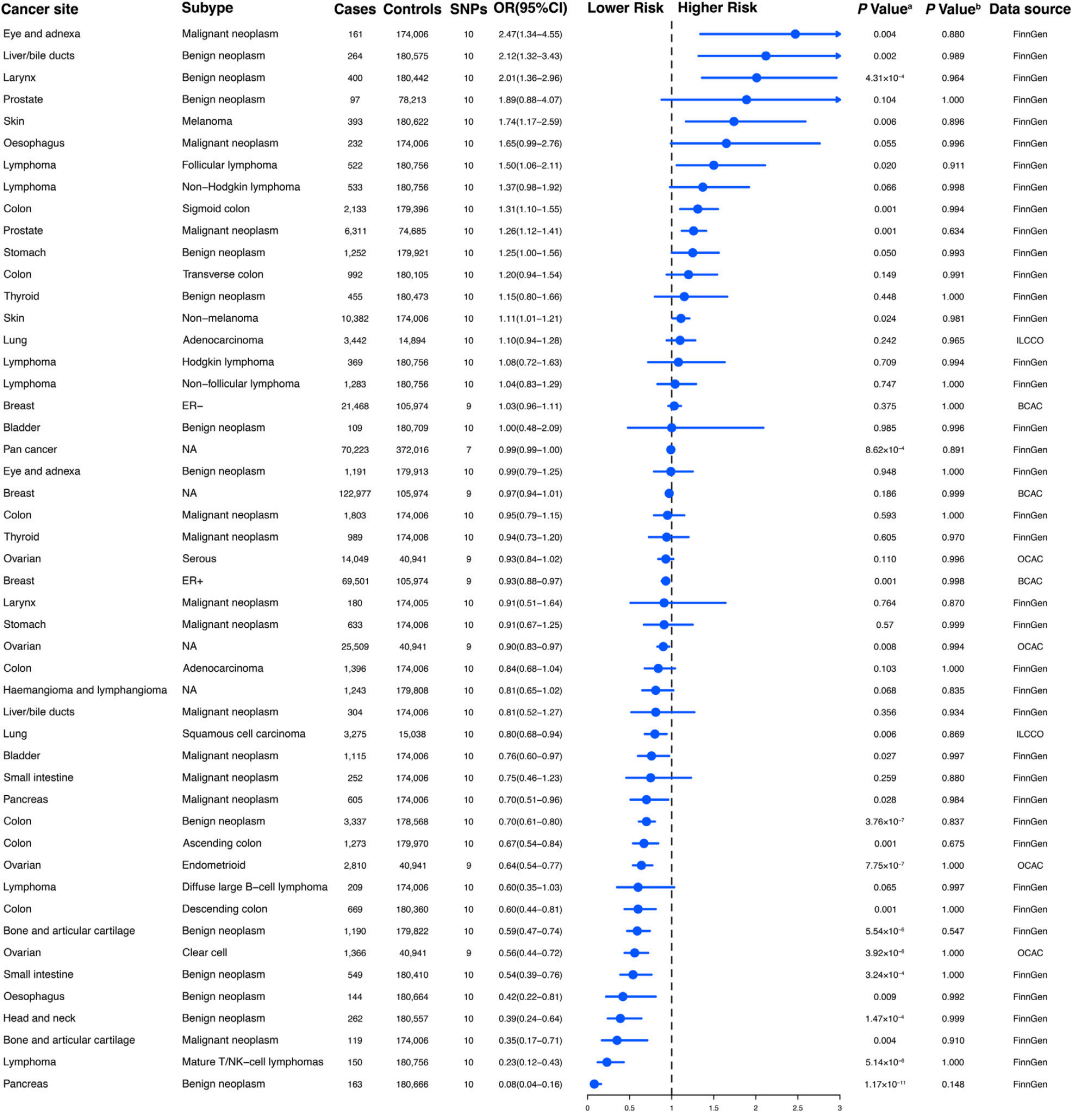
Conceptual framework of the Mendelian randomization study. The overarching objective of this study is to utilize genetic variants as instrumental variables (IVs) to estimate the unbiased causal relationship between regular glucosamine use and cancer and non-neoplastic disease risk. Toward this end, the association of IVs with both regular glucosamine use and cancer/non-neoplastic disease risk is leveraged to estimate the corresponding association between regular glucosamine use and these outcomes.

1. **Relevance:** The initial assumption for MR analyses is that the genetic instruments exhibit an association with the exposure of interest.
2. **Exchangeability:** Genetic instruments are independent of any and all confounders in the exposure-outcome association.
3. **Exclusion restriction:** Genetic instruments remain uncorrelated with the outcome when considering the exposures and any potential confounding variables (Figure 1).

### Study populations

We obtained summary statistics for habitual glucosamine consumption from the UK Biobank (UKB), which provided sufficiently powered genome-wide association study (GWAS) data. The cohort completed a touch screen questionnaire to gather data on regular glucosamine use. Our analysis focused on the 360,016 participants who reported taking glucosamine. MR analysis included a comprehensive set of SNPs that can be found in Supplementary Table S2. We obtained publicly available summary-level data for lung, breast, and ovarian cancer from the International Lung Cancer Consortium (11,348 cases and 15,861 controls) (Wang et al., 2014), the Breast Cancer Association Consortium (122,977 cases and 105,974 controls) (Michailidou et al., 2017), and the Ovarian Cancer Association Consortium (25,509 cases and 40,941 controls) (Phelan et al., 2017). The consortia’s participants were exclusively of European ancestry, hailing from European and North American countries along with Australia. The lung cancer consortium had participants of both genders, while the breast and ovarian cancer consortia only included women. The data were extracted from the consortia utilizing the MR-Base platform (Hemani et al., 2018). Genetic associations of 39 site-specific cancers, pan-cancer, and 14 non-neoplastic diseases were estimated in the most recently available R7 data release from the FinnGen consortium. The study was limited to individuals of European ancestry (https://www.finngen.fi/en/access_results (accessed on 15 May 2023)). FinnGen is a large public-private genome research project that collects and analyses genome and health data from Finnish biobanks and digital health record data from Finnish health registries. Its original phenotype definition mainly uses the International Classification of Diseases and Anatomical Chemical Therapeutic classification codes (Kurki et al., 2023). We extracted GWAS summary statistics for three psychiatric diseases, namely, bipolar disorder, anorexia nervosa, and autism spectrum disorder, from the Psychiatric Genomics Consortium website (https://www.med.unc.edu/pgc/results-and-downloads/(accessed on 15 May 2023)). As the largest consortium in the field of psychiatry, PGC has conducted influential meta- and mega-analyses of genome-wide genomic data for mental disorders. We obtained GWAS summary statistics for multiple sclerosis (MS) by using the summary statistics of the discovery cohorts of the latest International Multiple Sclerosis Genetics Consortium (IMSGC), which included 14,802 cases and 26,703 controls. The original publication provides a comprehensive description of demographic characteristics, MS case ascertainment, and eligibility criteria for the meta-analysis. Finally, we sourced GWAS summary statistics for Parkinson’s disease from the International Parkinson’s Disease Genomics Consortium (IPDGC), which involved 33,674 cases and 449,056 controls.

### Sample independence

**FIGURE 2:**
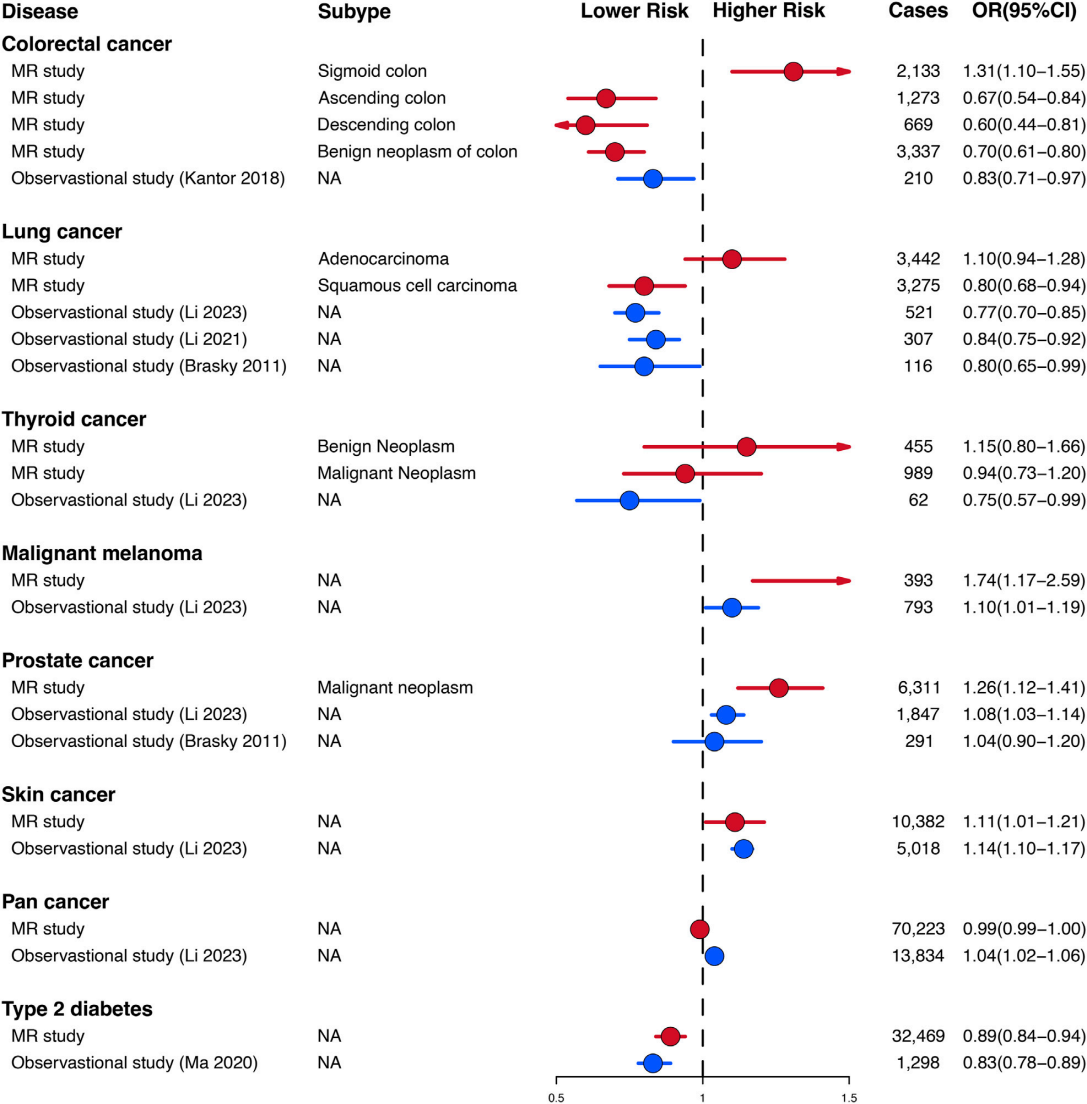
The association between genetically regular glucosamine use and risk of site-specific cancers. SNP, single-nucleotide polymorphism; OR, odds ratio; CI, confidence interval; NA, not applicable; ER, estrogen receptor; ILCCO, International Lung Cancer Consortium; BCAC, Breast Cancer Association Consortium; OCAC, Ovarian Cancer Association Consortium. **(A)** *p*-value for the association between genetically regular glucosamine use and cancer risk was estimated using random-effects inverse-variance weighting. **(B)** *p*-value for assessing heterogeneity among single-nucleotide polymorphisms within the instrumental variable.

**FIGURE 3:**
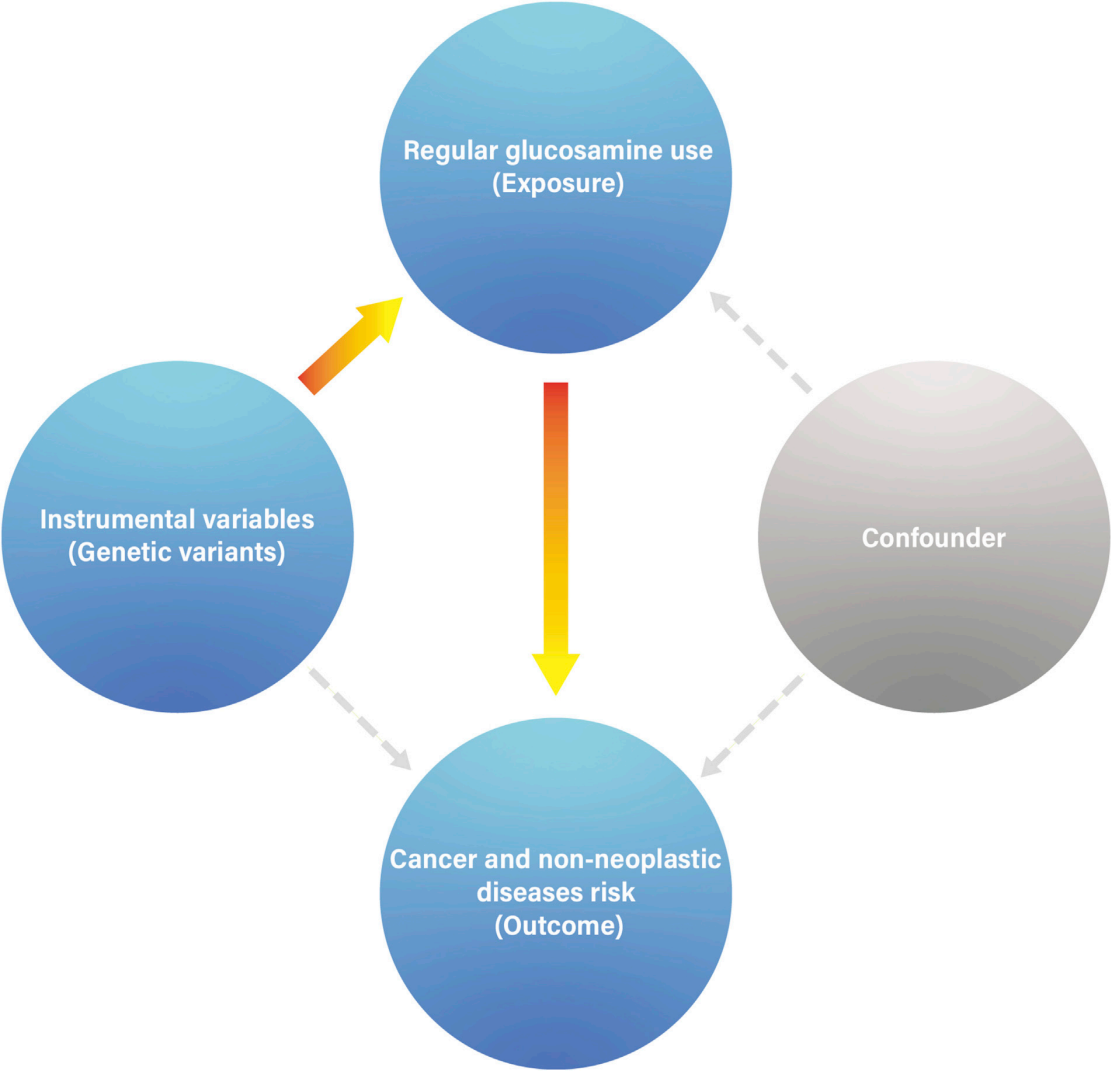
The association between genetically regular glucosamine use and risk of non-neoplastic diseases. SNP, single-nucleotide polymorphism; OR, odds ratio; CI, confidence interval; NA, not applicable; IMSGC, International Multiple Sclerosis Genetics Consortium; PGC, Psychiatric Genomics Consortium; IPDGC, International Parkinson’s Disease Genomics Consortium. **(A)** *p*-value for the association between genetically regular glucosamine use and cancer risk was estimated using random-effects inverse-variance weighting. **(B)** *p*-value for assessing heterogeneity among single-nucleotide polymorphisms within the instrumental variable.

In order to reduce the likelihood of population stratification bias, the GWASs for both cancer and non-cancerous diseases were confined to individuals with European ancestry, akin to the glucosamine GWAS. It is worth emphasizing that neither the UK Biobank nor the FinnGen study were major parts of the largest GWAS study, preventing the inclusion of overlapping samples that can increase weak instrument bias in MR analyses. Figures 2, 3 provide detailed information on pertinent characteristics of each cancer-specific and non-neoplastic disease GWAS, such as data sources and sample sizes. In summary, we obtained available GWAS data for 42 cancers and 20 non-neoplastic diseases as the primary outcomes of interest. Our analysis utilized summary-level GWAS data for both cancer and non-neoplastic ailments, wherein population details and quality control procedures were previously elucidated.

### Genetic instrument construction

To fulfill the initial MR assumption that necessitates the genetic instruments (SNP) to be robustly linked with the exposure (glucosamine), we included all relevant single-nucleotide variations identified in each GWAS as having reached the selection threshold of *p* < 5 × 10^−8^ and being uncorrelated (10,000 kilobase pairs apart and *R* ^2^ ≤ 001). Single nucleotide variant (SNV) effects and their corresponding standard errors were acquired from both the exposure and outcome GWASs. In order to prevent potential confounding, we meticulously assessed each genetic instrument’s SNP in the PhenoScanner GWAS database for prior associations (*p* < 5 × 10^−8^) with plausible confounders. To satisfy the assumption that requires genetic instruments to only affect the outcome through exposure, the analysis of cardiovascular diseases in the outcome was not included. Cohesive exposure and outcome data were harmonized, while palindromic SNPs with intermediate allele frequencies were removed. The F parameter was measured to assess instrument strength. Steiger filtering was adopted on the harmonized data to detect and exclude any SNPs exhibiting reverse causation with the test metric. Notably, the variance of the outcome observed surpassed the variance of the exposure explained by the SNPs.

### Mendelian randomization analyses

To evaluate the potential association between regular glucosamine use and risk of cancer and non-neoplastic diseases, we performed a primary analysis utilizing a two-sample Mendelian randomization approach that relied on inverse-variance weighting (IVW). Our methodology adhered to previously outlined protocols (Lawlor et al., 2008). In assessing the impact of each variant, we employed the Wald ratio method. Combining these individual MR assessments through random-effect inverse-variance meta-analysis allowed us to quantify the influence of a one standard deviation (SD) increase in standardized natural log-transformed regular glucosamine use on the risk of cancer and non-neoplastic disease.

To satisfy the second assumption of Mendelian randomization, we took great care to ensure that the genetic variants employed in our study were not associated with phenotypes that could potentially confound the exposure–outcome relationship. As part of a sensitivity analysis, we performed MR calculations by strategically excluding variants that were linked to potential confounders. To accomplish this, we utilized PhenoScanner to identify genetic variants related to GWAS traits that could introduce horizontal pleiotropy or function as confounders, specifically associating variants, for each glucosamine-related single nucleotide polymorphism (SNP) used as an instrument. During this search, we established positive associations when the GWAS *p*-value of the variant for a given trait was below the nominal *p*-value Bonferroni-corrected for the number of genetic variants (*p* < 0.05/10 = 0.005).

The third Mendelian randomization assumption necessitates that the genetic variants are not affiliated with the outcome through pathways separate from the exposure of interest, commonly referred to as the exclusion restriction assumption (Kaplan et al., 1985). Horizontal pleiotropy represents a situation in which this assumption is violated. To verify this assumption, several techniques that accounted for potential pleiotropic effects were employed. First, we assessed the heterogeneity of the SNPs utilized as instruments and calculated MR estimates by removing SNPs that appeared as outliers (Zhou, 1984). Following this, MR-Egger regression was applied to adjust for any possible unmeasured pleiotropy (Chevrel and Gueraud, 1974). The approach involved employing a weighted linear regression of the SNP (cancer and non-neoplastic diseases) susceptibility on the SNP (glucosamine) associations. This method enabled estimating the intercept as an indicator of the average pleiotropic effect and produced a slope coefficient representing a pleiotropy-robust MR estimate. By weakening the exclusion restriction assumption, MR-Egger was able to ascertain that the association of each variant with glucosamine was not tied to its pleiotropic effect (referred to as the InSIDE assumption). Furthermore, a weighted median analysis was conducted, entailing the weighting of individual MR estimates according to their precision (Lalonde, 1974). This strategy is founded on the premise that estimates derived from SNPs lacking pleiotropic effects are more prone to converging toward the median, whereas the introduction of pleiotropy can engender heterogeneity and lead to relative outliers. Reliable outcomes in this method are contingent on pleiotropic effects not exceeding 50% of the total weight. An alternative approach relying on a mode-based estimate instead of the median was also employed, which could accommodate even the majority of SNPs exhibiting pleiotropy (Smialek, 1969). To evaluate the robustness of our results, we conducted a leave-one-out analysis. Specifically, we excluded one single-nucleotide polymorphism at a time and performed an inverse-variance weighting analysis on the remaining SNPs to assess the impact of individual SNPs on the overall findings. This rigorous analytical approach scrutinizes the dependability of our results and provides further support for the validity and reliability of our conclusions. The implementation of various sensitivity analyses, with distinct underlying assumptions, contributed to our assurance that our conclusions were unlikely to be biased by pleiotropy.

The MendelianRandomization R package was utilized, with its default parameters, to generate four distinct MR estimates (IVW, weighted median, random-effects MR-Egger, and weighted mode) in the primary analysis, encompassing all SNPs and in sensitivity analyses that excluded SNPs connected to confounders. Of note, the IVW and MR-Egger methods employed the “random” model due to the presence of heterogeneity in our genetic instruments. In particular, the global examination identifies horizontal pleiotropy among MR instruments, the outlier assessment rectifies horizontal pleiotropy through the elimination of outliers, and the distortion analysis recognizes noteworthy distortion in causal estimates before and after the removal of outliers.

### Ethics

No ethical approval was required for the present study because all data sources were based on publicly available summary-level data. All these studies were approved by the relevant institutional review committees.

### Role of the funding source

The funding sources had no role in study design; in the collection, analysis, and interpretation of data; in the writing of the report; or in the decision to submit the article for publication.

## Results

### Causal effects of habitual glucosamine consumption on risk of site-specific cancers

To establish genetic instruments for habitual glucosamine consumption, we discerned ten distinct SNPs. These genetic instruments were measured for their strength using F-statistics, with any value exceeding 10 providing substantial evidence concerning the efficacy of the identified SNP as a strong instrument (Supplementary Table S2). We acquired summary data for genetic instruments associated with 49 distinct cancer types, including 378,284 cases and 533,969 controls (Figure 2). The number of available SNPs varied among the diseases, with a median of 10 (ranging from 3 to 10).

According to the results of the present study, habitual glucosamine consumption was found to be associated with increased odds ratios and confidence intervals of disease in the context of 18 primary cancers out of the total of 30 cancers examined (*p* < 0.05). The specific cancers showing significantly higher odds ratios were malignant neoplasm of the eye and adnexa (2.47 [1.34–4.55]), benign neoplasm of the liver/bile ducts (2.12 [1.32–3.43]), benign neoplasm of the larynx (2.01 [1.36–2.96]), melanoma (1.74 [1.17–2.59]), follicular lymphoma (1.50 [1.06–2.11]), sigmoid colon cancer (1.31 [1.10–1.55]), malignant neoplasm of the prostate (1.26 [1.12–1.41]), and non-melanoma skin cancer (1.11 [1.01–1.21]) (Figure 2). Conversely, habitual glucosamine intake was correlated with decreased odds ratios (95% confidence intervals) for 18 cancers (*p* < 0.05). These cancers include benign neoplasm of the pancreas (0.08 [0.04–0.16]), mature T/NK-cell lymphomas (0.23 [0.12–0.43]), malignant neoplasm of bone and articular cartilage (0.35 [0.17–0.71]), benign neoplasm of the head and neck (0.39 [0.24–0.64]), benign neoplasm of the esophagus (0.42 [0.22–0.81]), benign neoplasm of the small intestine (0.54 [0.39–0.76]), clear cell ovarian cancer (0.56 [0.44–0.72]), benign neoplasm of bone and articular cartilage (0.59 [0.47–0.74]), descending colon cancer (0.60 [0.44–0.81]), endometrioid ovarian cancer (0.64 [0.54–0.77]), ascending colon cancer (0.67 [0.54–0.84]), benign neoplasm of colon cancer (0.70 [0.61–0.80]), malignant neoplasm of the pancreas (0.70 [0.51–0.96]), malignant neoplasm of the bladder (0.76 [0.60–0.97]), squamous cell lung carcinoma (0.80 [0.68–0.94]), ovarian cancer (0.90 [0.83–0.97]), and estrogen receptor-positive breast cancer (0.93 [0.88–0.97]). The strongest evidence of association was observed for malignant neoplasm of the eye and adnexa, benign neoplasm of the liver/bile ducts, benign neoplasm of the larynx, benign neoplasm of the pancreas, mature T/NK-cell lymphomas, malignant neoplasm of bone and articular cartilage, benign neoplasm of the head and neck, and benign neoplasm of the esophagus. These findings imply that the nuanced nature of glucosamine’s impact on cancer risk may rest on the distinct classification of cancer, as varied depths and characters of effect were observed.

### Causal effects of habitual glucosamine consumption on risk of non-neoplastic diseases

We obtained summary data for the genetic instruments related to 20 non-neoplastic diseases, representing 292,270 cases and 842,829 controls (Figure 3). The available number of SNPs varied across the different diseases, with a median of 10 (with a minimum of 3 and a maximum of 10).

Habitual glucosamine consumption was correlated with elevated odds ratios (95% confidence intervals) of illness for six of 14 primary non-neoplastic diseases (*p* < 0.05): autoimmune thyroiditis (2.47 [1.49–4.08]), autoimmune hyperthyroidism (1.93 [1.17–3.18]), chronic sinusitis (1.28 [1.17–1.40]), atopic dermatitis (1.24 [1.11–1.39]), asthma (1.20 [1.12–1.28]), and bipolar disorder (1.16 [1.05–1.27]) (Figure 3). In contrast, habitual glucosamine consumption was associated with a decrease in odds ratios (95% confidence intervals) intervals for eight non-neoplastic diseases (*p* < 0.05), including interstitial lung disease (0.57 [0.48–0.68]), inflammatory bowel disease (0.73 [0.66–0.82]), multiple sclerosis (0.73 [0.66–0.82]), anorexia nervosa (0.75 [0.64–0.89]), type 1 diabetes (0.75 [0.67–0.85]), autism spectrum disorder (0.84 [0.77–0.91]), type 2 diabetes (0.89 [0.84–0.94]), and gastroesophageal reflux disease (0.92 [0.85–0.99]). The most robust evidence of association was found for interstitial lung disease and autoimmune thyroiditis. Interestingly, while the association between habitual glucosamine consumption and risk of autoimmune thyroiditis and autoimmune hyperthyroidism demonstrated elevated odds ratios as previously described, the presence of broad confidence intervals suggests significant variability.

### Comparison with prospective observational studies

**FIGURE 4:**
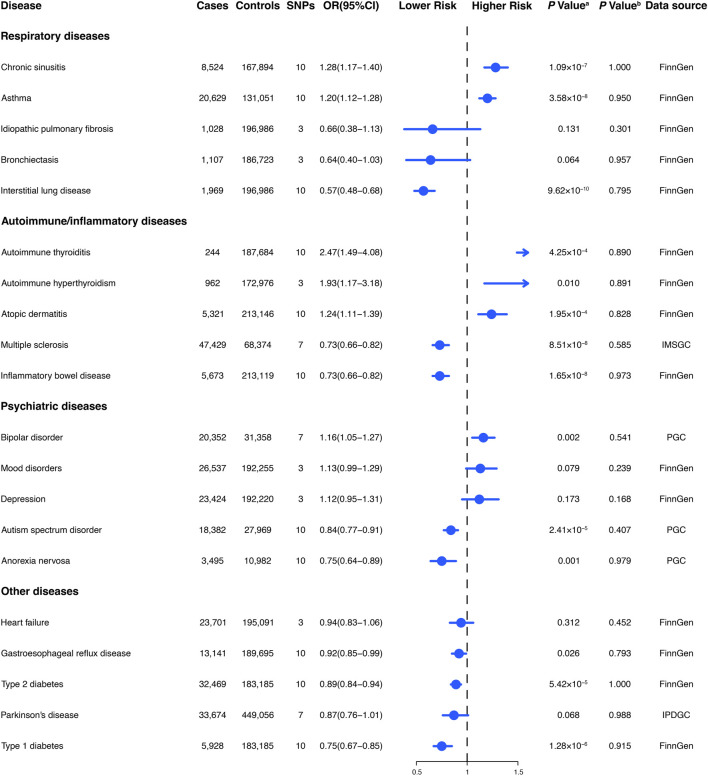
Comparison of Mendelian randomization investigation and prospective observational studies of the association between habitual glucosamine consumption and the risk of disease.

The genetic findings of our study on the relationship between habitual glucosamine consumption and malignant melanoma, prostate cancer, skin cancer, and type 2 diabetes are consistent in direction and magnitude with estimates from prospective observational studies (Figure 4). Notably, our study uncovered significant disparities in certain findings when compared to the observational prospective studies. Specifically, our genetic evaluations demonstrated that glucosamine intake is positively linked to an elevated risk of sigmoid colon cancer (1.31 [1.10–1.55]), lung adenocarcinoma (1.10 [0.94–1.28]), and benign neoplasm of the thyroid (1.15 [0.80–1.66]).

### Sensitivity analyses

We conducted sensitivity analyses to assess the likely influence of confounding via pleiotropic pathways on our outcomes. The results obtained with the weighted median and MR-Egger approaches were found to be generally consistent with those of the primary analysis (inverse-variance weighted) (Supplementary Tables S5, S6). Our analyses suggest minimal evidence for heterogeneity (*P* heterogeneity >0.05) or pleiotropy (MR-Egger intercept test). Nevertheless, we must acknowledge that the MR-Egger analyses may have lacked statistical power, given the wide confidence intervals observed in the estimated odds ratios. We conducted a leave-one-out analysis to scrutinize the reliability of our findings. To this end, we excluded individual single-nucleotide polymorphisms in turn and performed inverse-variance weighting analysis on the remaining SNPs to evaluate their robustness. Our rigorous examination detected no instrumental variables that significantly deviated from expectations, attesting to the credibility of our results (Supplementary Tables S7, S8).

## Discussion

In this investigation, we show that genetically predicted habitual glucosamine consumption is associated with heightened susceptibility to a diverse spectrum of cancerous as well as non-neoplastic diseases, exerting dichotomous effects on disease trajectories. Casting doubt on the purported purely beneficial association between glucosamine ingestion and prevention of neoplastic and non-neoplastic diseases, the present investigation instead highlighted the potential for unfavorable outcomes. Considering the random distribution of genotypes among the general populace with regard to environmental factors and lifestyle, in addition to the unvarying characteristics of germline genotypes, our findings are presumed to be less prone to confounding and reverse causation than those derived from observational studies. Our findings indicate that habitual glucosamine consumption is associated with an increased risk of some cancers, including malignant neoplasm of the eye and adnexa, benign neoplasm of the liver/bile ducts, benign neoplasm of the larynx, melanoma, and follicular lymphoma. Moreover, genetically predicted glucosamine intake exhibited a positive correlation with the risk of certain non-neoplastic disorders, such as autoimmune thyroiditis, autoimmune hyperthyroidism, chronic sinusitis, atopic dermatitis, asthma, and bipolar disorder. The association between genetically determined glucosamine intake and cancer risk may vary across different cancer types. Intriguingly, certain tumors, such as malignant neoplasm of the eye and adnexa (2.47 [1.34–4.55]), benign neoplasm of the liver/bile ducts (2.12 [1.32–3.43]), benign neoplasm of the larynx (2.01 [1.36–2.96]), melanoma (1.74 [1.17–2.59]), and follicular lymphoma (1.50 [1.06–2.11]), exhibited not only increased odds ratios as previously described but also wide confidence intervals, indicating considerable variability. Variability is manifested in the dissimilarities between the benign and malignant character of certain tumors. For the esophagus, the odds ratios (95% confidence intervals) were (0.42 [0.22–0.81]) for benign neoplasm compared with (1.65 [0.99–2.76]) for malignant neoplasm. In contrast, for bone and articular cartilage, the odds ratios (95% confidence intervals) were (0.59 [0.47–0.74]) for benign neoplasm and (0.35 [0.17–0.71]) for malignant neoplasm. This finding put forth a plausible sequence of stage-specific molecular modifications that steer the discrete phases of tumor evolution induced by regular intake of glucosamine. Moreover, the observation hints at conceivable divergences in the underlying mechanisms governing tumor progression at distinct developmental stages. Significant heterogeneity was observed in the histological subtypes of cancers. For example, the odds ratios (95% confidence intervals) for follicular lymphoma were (1.50 [1.06–2.11]) compared with (0.23 [0.12–0.43]) for mature T/NK-cell lymphoma. Substantial variability was also observed within tissue sites. The odds ratio (95% confidence intervals)) for sigmoid colon cancer was (1.31 [1.10–1.55]) and transverse colon cancer was (1.20 [0.94–1.54]) compared with (0.67 [0.54–0.84]) for ascending colon cancer and (0.60 [0.44–0.81]) for descending colon cancer. In contrast, for ovarian cancer, the odds ratios (95% confidence intervals) were (0.56 [0.44–0.72]) for clear cell ovarian cancer and (0.64 [0.54–0.77]) for endometrioid ovarian cancer. Furthermore, our study identified potential associations indicating an increased risk of certain diseases, including benign neoplasm of the prostate, malignant neoplasm of the esophagus, and non-Hodgkin lymphoma, although these were not statistically significant. These findings warrant further investigation to determine their significance and clinical implications. Our results indicate that timely oncotyping could facilitate the identification of individuals who are vulnerable to potential hazards associated with habitual glucosamine consumption.

Nevertheless, certain findings from our study display notable disparities when compared to preceding observational studies. The results of analyses conducted on the Cancer Prevention Study II Nutrition Cohort, which updated glucosamine use data every 2 years for both cohorts and confirmed colorectal cancer cases through medical records, demonstrated that there was an association between the consumption of glucosamine and a 17% (OR 0.83, 95% CI: 0.71–0.97) reduced risk of colorectal cancer (Kantor et al., 2018). In contrast with the findings presented in the preceding observational study, our genetic assessments illustrate that glucosamine intake is positively correlated with a 31% increase in sigmoid colon cancer (OR 1.31, 95% CI: 1.10–1.55), a 10% increase in lung adenocarcinoma risk (OR 1.10, 95% CI: 0.94–1.28) and a 15% increase in the risk of benign neoplasm of the thyroid (OR 1.15, 95% CI: 0.80–1.66). Our observations have furthered the understanding of the various effects that glucosamine use has on different tumor properties, which were not apparent in observational studies.

Remarkably, our findings show that the administration of glucosamine displayed adverse effects on malignant thyroid tumors by decreasing their risk (OR 0.94, 95% CI: 0.73–1.20), in agreement with Li et al.’s research (OR 0.75, 95% CI: 0.57–0.99), while simultaneously demonstrating a contrary impact on benign thyroid tumors by elevating their associated risk (OR 1.15, 95% CI: 0.80–1.66) (Hemani et al., 2018). This observation also underscores the significance of taking into account the particular subtype of tumor in evaluating the potential impact and hazards associated with the utilization of glucosamine in future observational studies.

Our genetic discoveries concur with estimates obtained from observational investigations that have analyzed the correlation between glucosamine utilization and the risk of squamous cell lung carcinoma, prostate cancer, skin cancer, malignant melanoma, and type 2 diabetes. Analyses based on the UK Biobank cohort, consisting of more than 500,000 subjects aged between 40 and 69 years recruited in the period of 2006–2010 in the United Kingdom, revealed that glucosamine intake was linked to a 10% (OR 1.10, 95% CI: 1.01–1.19) higher risk of malignant melanoma and a 14% (OR 1.14, 95% CI: 1.10–1.17) increased risk of skin cancer (Hemani et al., 2018). Through analyzing data from the UK Biobank cohort study of 404,508 participants, Ma et al. discovered that the consumption of glucosamine may have a protective association with the risk of type 2 diabetes (OR 0.83, 95% CI: 0.78–0.89) (Ma et al., 2020). An investigation of the VITamins And Lifestyle (VITAL) cohort study, which evaluated 77,719 inhabitants of western Washington State aged 50–76 years during the period spanning October 2000 to December 2002, disclosed that the intake of glucosamine was linked with a 20% (OR 0.80, 95% CI: 0.65–0.99) reduction in lung cancer risk and a 4% (OR 1.04, 95% CI: 0.90–1.20) increase in prostate cancer risk (Brasky et al., 2011a; Brasky et al., 2011b). An examination of the UK Biobank cohort, which enrolled more than 500,000 subjects aged between 40 and 69 years in 2006–2010, revealed that the consumption of glucosamine was associated with a 16% decreased risk of lung cancer (OR 0.84, 95% CI: 0.75–0.92) (Kaplan et al., 1985; Kurki et al., 2023). It is of paramount significance to note that our genetic assessments with regard to malignant melanoma (OR 1.74, 95% CI: 1.17–2.59), prostate cancer (OR 1.26, 95% CI: 1.12–1.41), and type 2 diabetes (OR 0.89, 95% CI:0.84–0.94) have demonstrated a higher degree of robustness when contrasted against these observational investigations.

The work of Suissa et al. has revealed a potential issue with observational studies that have reported benefits associated with glucosamine usage, such as lowered mortality rates and reduced cancer incidence. Specifically, the authors identify collider stratification as a source of selection bias that may impact the validity of these findings (Suissa et al., 2022). The phenomenon known as collider bias, or collider-stratification bias, arises in observational research when the study group is chosen based on a criterion—referred to as the collider—that is linked with the exposure being investigated and shares risk factors with the outcome under scrutiny. Collider-stratification bias has the potential to create a spurious association between the exposure and outcome, leading to erroneous conclusions. Furthermore, it can generate an association where none exists or even reverse the direction of an actual association, thereby creating a paradoxical relationship that makes a harmful exposure seem protective (Hernan et al., 2004; Hernandez-Diaz et al., 2006). In addition, Li et al. reported a stronger association between glucosamine use and a lower risk of colorectal cancer in participants without screening, but no significant association was observed among screened individuals. This finding may be attributed to detection bias, where individuals taking glucosamine and undergoing colorectal cancer screening are more likely to have early detection of colorectal cancer (Hemani et al., 2018). Due to the arbitrary distribution of genotypes in the broader populace regarding environmental factors and way of life and the immutable quality of germline genotypes, compared to observational investigations, outcomes derived from this MR study should possess a reduced susceptibility to confounding and reverse causality.

A wealth of scientific research has explored the anticancer mechanisms that reinforce the seemingly indomitable position of regular glucosamine use as a prominent anticancer agent. Glucosamine plays multiple pivotal roles in various cellular processes, such as altering the composition of uracil and adenine nucleotides (Plagemann and Erbe, 1973; Decker and Keppler, 1974; Holstege et al., 1982), disrupting cell membrane systems (Bosmann, 1971; Molnar and Bekesi, 1972; Friedman et al., 1977; Friedman et al., 1985), inducing autophagic cell death (Marshall et al., 2004; Hwang and Baek, 2010; Shintani et al., 2010; Carames et al., 2013; Jiang et al., 2014; Yu et al., 2017), inhibiting ubiquitin proteasome (Su et al., 2000; Liu et al., 2004; Liu et al., 2011) and STAT-3 signaling pathways (Gewinner et al., 2004; Chesnokov et al., 2009; Rebe et al., 2013; Wang et al., 2017), suppressing HIF-1 activity (Gaben et al., 2004; Jung et al., 2012; Song et al., 2014), and exhibiting antioxidant (Xing et al., 2006; Yan et al., 2007; Mendis et al., 2008; Valvason et al., 2008; Jamialahmadi et al., 2014), anti-angiogenic (Xu et al., 2012), immunostimulatory, and anti-inflammatory (Largo et al., 2003; Chan et al., 2005; Yomogida et al., 2008; Xing et al., 2011; Azuma et al., 2015; Chou et al., 2015; Someya et al., 2016; Leopizzi et al., 2017; Yamagishi et al., 2017) effects. Notwithstanding, there exist certain biological indications that lend credence to the probability of the amplified risk that we have witnessed in instances of prostate cancer. Li Feng et al. reported habitual glucosamine consumption has the potential to trigger an increase in the levels of insulin-like growth factor-I, known to be a contributing factor to the risk of prostate cancer (Travis et al., 2016; Feng et al., 2020).

However, the observed outcomes have initiated a reevaluation and reconsideration of the potential relationship between the habitual consumption of supplements, such as glucosamine, and the incidence of various diseases. A recent study conducted by Lin and colleagues presents a pertinent example of the complex interplay between dietary supplements and cancer biology. Chondroitin-4-sulfate (CHSA), which is typically co-administered with glucosamine as a dietary supplement for osteoarthritis management, has been found to selectively enhance the tumor growth potential of BRAF V600E-expressing human melanoma cells in xenograft mice derived from patients or cell lines, as well as to impart resistance to BRAF inhibitors (Lin et al., 2018). Similarly, Le Gal et al. reported the administration of antioxidants N-acetylcysteine and vitamin E via supplementation inhibited p53 expression in murine models of lung cancer, resulting in an escalation of lung tumor progression and a decrease in survival rates (Sayin et al., 2014). The biological effects of regular glucosamine intake in relation to genetic backgrounds have not been thoroughly investigated, and the pathogenic connections between glucosamine use and particular oncogenic mutations remain unclear. Thus, further studies are required to explore the potential mechanisms underlying how dietary supplements may promote oncogenesis. Such investigations could provide valuable insights into selecting appropriate regular glucosamine intake with minimal cancer risk based on individual genetic profiles. According to Li et al., skin cancer was identified as the predominant cause for the elevated overall cancer risk after certain cancer types were censored in the sensitivity analysis (Hemani et al., 2018). The observed dualistic impact of glucosamine intake on tumorigenesis risk can be attributed to differential mechanisms operative at the cellular and molecular biology levels of tumors during distinct stages of tumor progression.

The present study elucidates the multifaceted interplay between regular glucosamine intake and the risk of cancer and non-neoplastic diseases, emphasizing the significance of prudent consideration and vigilant utilization in clinical and home settings. Our investigation does not support a unidirectional beneficial association between glucosamine usage and the prevention of neoplastic and non-neoplastic diseases. Instead, our results suggest the possibility of detrimental repercussions and divergent effects on the trajectory of disease progression due to habitual intake of glucosamine. Nevertheless, it should be acknowledged that our observations do not preclude the existence of nuanced consequences that require further investigation. The mechanisms underlying the potential long-term impacts of glucosamine consumption on the pathogenesis and evolution of both neoplastic and non-neoplastic conditions are not yet well-defined, warranting further investigation. As a result, our findings suggest that using glucosamine as a preventative measure to diminish the likelihood of cancer or non-neoplastic disorders cannot be endorsed.

The strengths of our study are manifold. Specifically, this investigation constitutes the inaugural large-scale analysis utilizing MR methodology to systematically examine the causal link between habitual glucosamine intake and the susceptibility to a range of cancers as well as non-neoplastic diseases. The study leveraged cancer GWASs featuring a composite sample of 912,253 individuals of European lineage (comprising 378,284 cancer instances and 533,969 controls) and non-neoplastic disease GWAS data encompassing 1,135,099 subjects of European ancestry (encompassing 292,270 cases and 842,829 controls). Additionally, to test for MR assumption violations, this MR analysis incorporated a range of reliable methods and sensitivity analyses. No discernible connection was observed between genetically predicted habitual glucosamine consumption and cancer/non-neoplastic diseases. In addition to scrutinizing potential sources of heterogeneity in the findings for site-specific cancers, we also perform a comparative analysis between genetic estimates and observations from prospective studies that evaluate the causal association between glucosamine consumption and the risk of noncommunicable diseases.

### Study limitations

Acknowledgment of certain limitations is warranted. First, this investigation scrutinized the impact of administering exogenous glucosamine, yet it seems improbable that this alone offers a comprehensive explanation for the influence of overall glucosamine levels in the body, encompassing the endogenous aspect. Second, the pool of genetic instruments available for investigating habitual glucosamine consumption is presently restricted, comprising a mere ten genetic variants. This circumstance may have repercussions on the ability to detect pleiotropy through the employment of MR-Egger methods—although none of our pleiotropy tests disclosed statistically significant infringements, these diagnostic assessments are liable to suffer from insufficient statistical power. Hence, it is necessary to identify additional instrumental variables associated with habitual glucosamine usage. Third, it should be recognized that our findings may not generalize comprehensively to all noncommunicable diseases, given the absence of data sharing among some studies. Nevertheless, the diseases under scrutiny in our primary investigations are likely responsible for more than 60% of mortalities across various age groups in the American adult population (accessed on 11 June 2023; https://www.cdc.gov/nchs/fastats/deaths.htm). Fourth, the genetic association estimations derived from study designs utilized in GWAS may have been impacted by bias, especially survival bias. It is plausible that if genetic variants were used to instrument regular glucosamine use and such usage increased disease risk and mortality before enrollment in a case-control study, an artificial defensive relationship between glucosamine use and disease incidence might require investigation. Fifth, the possibility of chance as an explanatory factor for some of the less robust results cannot be entirely discounted. Sixth, although MR analysis can provide insight into the lifetime impact of habitual glucosamine use on cancer and non-neoplastic disorders, the clinical significance of such estimates regarding age-specific interventions is limited. To address this limitation, it would be beneficial to conduct future MR analyses with a gender- or age-specific focus, utilizing larger sample sizes in order to provide more meaningful results. Finally, the study’s sample population was limited to individuals of European ethnicity, causing the transferability of the results to other ethnic groups to remain indeterminate.

## Conclusion

Through the application of Mendelian randomization analyses, we observe an intriguing deviation from the conventional biological understanding, revealing the Janus-faced role of habitual glucosamine ingestion on the risk of disease. Our study uncovers a novel revelation that contradicts the widely held perception of a solely protective correlation between genetically proxied glucosamine consumption and the risk of cancer and non-neoplastic diseases. Therefore, endorsing habitual glucosamine consumption as a prophylactic measure against both neoplastic and non-neoplastic diseases cannot be supported. More crucially, it is evident that a comprehensive evaluation of the safety of glucosamine intake through clinical trials, incorporating suitable follow-up measures, is urgently warranted.

## Data Availability

The original contributions presented in the study are included in the article/Supplementary Material; further inquiries can be directed to the corresponding authors.
