# Correction: Association between genetically proxied glucosamine and risk of cancer and non-neoplastic disease: a mendelian randomization study

**DOI:** 10.3389/fgene.2025.1660021

**Published:** 2025-08-07

**Authors:** Yingtong Wu, Yinggang Che, Yong Zhang, Yanlu Xiong, Chen Shu, Jun Jiang, Gaozhi Li, Lin Guo, Tianyun Qiao, Shuwen Li, Ou Li, Ning Chang, Xinxin Zhang, Minzhe Zhang, Dan Qiu, Hangtian Xi, Jinggeng Li, Xiangxiang Chen, Mingxiang Ye, Jian Zhang

**Affiliations:** ^1^ Department of Pulmonary Medicine, Xi’an People’s Hospital, Xi’an, China; ^2^ First Sanatorium, Air Force Healthcare Center for Special Services, Hangzhou, China; ^3^ Department of Thoracic Surgery, Tangdu Hospital, Air-Force Medical University, Xi’an, China; ^4^ Department of Health Service, Air-Force Medical University, Xi’an, China; ^5^ 94498th Unit of the People’s Liberation Army of China, Nanyang, China; ^6^ Department of Obstetrics and Gynecology, Tangdu Hospital, Air-Force Medical University, Xi’an, China; ^7^ College of pulmonary and Critical Care Medicine, The 8th Medical Centre of Chinese PLA General Hospital, Beijing, China; ^8^ Department of Respiratory Medicine, Jinling Hospital, Nanjing University School of Medicine, Nanjing, China

**Keywords:** glucosamine, cancer risk, mendelian randomization, single-nucleotide polymorphisms, causality

In the published article, there was an error in **Affiliation** 1. Instead of “Department of Pulmonary and Critical Care Medicine, Xijing Hospital, Air-Force Medical University, Xi’an, China”, it should be “Department of Pulmonary Medicine, Xi’an People’s Hospital, Xi’an, China”.

The original article has been updated.

