# Supplementary material for: Association between genetically proxied glucosamine and risk of cancer and non-neoplastic disease: A Mendelian randomization study
Source: Front Genet. 2024 Jun 27;15:1293668. doi: 10.3389/fgene.2024.1293668 (PMC11236616; doi:10.3389/fgene.2024.1293668)
Supplement: Supplementary file 3 [file Table1.DOCX]

**Table S1**: Self-inspection results of STROBE-MR checklist of recommended items to address in reports of Mendelian randomization studies.

| **Item No.** | **Section** | **Checklist item** | **Page No.** | **Relevant text from manuscript** |
| --- | --- | --- | --- | --- |
| 1 | **TITLE and ABSTRACT** | Indicate Mendelian randomization (MR) as the study’s design in the title and/or the abstract if that is a main purpose of the study | 1-4 | The Janus-faced role of glucosamine in cancer and non-neoplastic disease: A Mendelian randomization study. |
|  | **INTRODUCTION** |  |  |  |
| 2 | **Background** | Explain the scientific background and rationale for the reported study. What is the exposure? Is a potential causal relationship between exposure and outcome plausible? Justify why MR is a helpful method to address the study question | 2 | The causal relationship and magnitude of the correlation between habitual glucosamine consumption and the occurrence of cancer and non-neoplastic diseases remain uncertain due to the susceptibility of observational studies to potential selection bias and reverse causation, which may limit the accuracy of the findings. The natural variation in genes that encode glucosamine targets can serve as proxies for the investigation of their impact on disease outcomes. |
| 3 | **Objectives** | State specific objectives clearly, including pre-specified causal hypotheses (if any). State that MR is a method that, under specific assumptions, intends to estimate causal effects | 2-4 | Taking into account the preceding knowledge, we formulated a hypothesis which postulated that regular glucosamine intake might be linked to the incidence of both cancer and non-neoplastic diseases. To establish an underlying causal relationship between habitual glucosamine consumption and the onset of such ailments, we conducted a two-sample Mendelian randomization (MR) analysis.  The MR approach is predicated on the principle that genetic variants are randomly inherited from each parent (known as the law of segregation assortment), and that alleles are transmitted independently of one another to offspring (known as the law of independent assortment). This methodological framework effectively circumvents the influence of environmental factors that may obscure the estimated relationship between the variables under study in the MR analysis. |
|  | **METHODS** |  |  |  |
| 4 | **Study design and data sources** | Present key elements of the study design early in the article. Consider including a table listing sources of data for all phases of the study. For each data source contributing to the analysis, describe the following: |  |  |
|  | a) | Setting: Describe the study design and the underlying population, if possible. Describe the setting, locations, and relevant dates, including periods of recruitment, exposure, follow-up, and data collection, when available. | 5-7 | Detailed information can be found in the Methods section of original paper. |
|  | b) | Participants: Give the eligibility criteria, and the sources and methods of selection of participants. Report the sample size, and whether any power or sample size calculations were carried out prior to the main analysis | 5-7 | Detailed information can be found in the Methods section of original paper. |
|  | c) | Describe measurement, quality control and selection of genetic variants | 7,8 | Detailed information can be found in the Methods section of original paper. |
|  | d) | For each exposure, outcome, and other relevant variables, describe methods of assessment and diagnostic criteria for diseases | 7 -10 | Detailed information can be found in the Methods section of original paper. |
|  | e) | Provide details of ethics committee approval and participant informed consent, if relevant | 7-10 | The data analyzed in this study is publicly available from existing, published GWASs and therefore the ethical approval and informed consent have been obtained by all original studies. |
| 5 | **Assumptions** | Explicitly state the three core IV assumptions for the main analysis (relevance, independence and exclusion restriction) as well assumptions for any additional or sensitivity analysis | 4,5 | The selection of IVs, the key to ensure the accuracy and robustness of the causal inferences, must meet MR's three key assumptions (Figure 1).  Three key assumptions of MR: (1) genetic variants must be associated with exposures; (2) genetic variants must not be associated with confounders; (3) genetic variants must affect outcomes only through exposures, not through other pathways. |
| 6 | **Statistical methods: main analysis** | Describe statistical methods and statistics used |  |  |
|  | a) | Describe how quantitative variables were handled in the analyses (i.e., scale, units, model) | 4-10 | Detailed information can be found in the Methods section of original paper. |
|  | b) | Describe how genetic variants were handled in the analyses and, if applicable, how their weights were selected | 7,8 | Genetic variants were handled in the analyses via three key stages. Initial screening excluded palindromic variants with minor allele frequencies exceeding 0.4. Thereafter, variants and their corresponding alleles were reconciled between the GWAS outcomes pertaining to exposure and outcome. In order to reduce the effects of linkage disequilibrium, independent SNPs located within 250 kb (using the European-based reference panel from the 1,000 Genome Projects) and possessing an LD r2 < 0.01 were identified, while maintaining a reasonable level of statistical significance. |
|  | c) | Describe the MR estimator (e.g. two-stage least squares, Wald ratio) and related statistics. Detail the included covariates and, in case of two-sample MR, whether the same covariate set was used for adjustment in the two samples | 8-10 | Sex and age are consistently included as covariates, although the specific list of covariates can vary in original GWASs. Additional information regarding the covariate selection can be retrieved from the primary investigations. |
|  | d) | Explain how missing data were addressed | No Applicable | No Applicable |
|  | e) | If applicable, indicate how multiple testing was addressed | No Applicable | No Applicable |
| 7 | **Assessment of assumptions** | Describe any methods or prior knowledge used to assess the assumptions or justify their validity | 8-10 | We selected variants that were strongly associated with the exposures at GWAS significance i.e. P< 5×10^-8^ to comply with assumption 1. We additionally used a large number of robust methods and sensitivity analyses, including adjustments for probable confounders, using different sets of IVs and exclusion of IVs that were associated with secondary traits, to probe into potential violations of assumptions 2 and 3.  Detailed information can be found in the Methods section of original paper. |
| 8 | **Sensitivity analyses and additional analyses** | Describe any sensitivity analyses or additional analyses performed (e.g. comparison of effect estimates from different approaches, independent replication, bias analytic techniques, validation of instruments, simulations) | 8-10 | All sensitivity analyses can be found in the Methods section of original paper. |
| 9 | **Software and pre-registration** |  |  |  |
|  | a) | Name statistical software and package(s), including version and settings used | 9,10 | MR were performed using the “TwoSampleMR” (version 0.5.7) and “MendelianRandomization” (version 0.7.0) packages in R (version 4.3.0). |
|  | b) | State whether the study protocol and details were pre-registered (as well as when and where) | No Applicable | This is a secondary analysis based on summary statistics from existing, published studies. The ethical approval and informed consent have been obtained by all original studies. |
|  | **RESULTS** |  |  |  |
| 10 | **Descriptive data** |  |  |  |
|  | a) | Report the numbers of individuals at each stage of included studies and reasons for exclusion. Consider use of a flow diagram | 5-7 | Detailed information can be found in the Methods section of original paper. |
|  | b) | Report summary statistics for phenotypic exposure(s), outcome(s), and other relevant variables (e.g. means, SDs, proportions) | 5-8 | The genetic instrument variables (IVs), typically single-nucleotide polymorphisms (SNPs), for habitual glucosamine consumption were retrieved from summary statistics derived from 361,194 participants of white-British ancestry that were publicly released by the Neale Lab (http://www.nealelab.is/uk-biobank)......  Detailed information can be found in the Methods section of original paper. |
|  | c) | If the data sources include meta-analyses of previous studies, provide the assessments of heterogeneity across these studies | No Applicable | No Applicable |
|  | d) | For two-sample MR:  i.  Provide justification of the similarity of the genetic variant-exposure associations between the exposure and outcome samples  ii.  Provide information on the number of individuals who overlap between the exposure and outcome studies | 7 | These GWAS sample populations needed to be predominantly of European descent and largely independent of each other |
| 11 | **Main results** |  |  |  |
|  | a) | Report the associations between genetic variant and exposure, and between genetic variant and outcome, preferably on an interpretable scale | 7-8 | The associations between all IVs used in our analyses and our exposures and outcomes are reported in supplementary S2-3 tables. |
|  | b) | Report MR estimates of the relationship between exposure and outcome, and the measures of uncertainty from the MR analysis, on an interpretable scale, such as odds ratio or relative risk per SD difference | 14-17  Fig 2-3 | The limited sample size may also prevent us from providing a sufficiently precise estimate as well as 95% confidence intervals for clinical practice. |
|  | c) | If relevant, consider translating estimates of relative risk into absolute risk for a meaningful time period | No Applicable | No Applicable |
|  | d) | Consider plots to visualize results (e.g. forest plot, scatterplot of associations between genetic variants and outcome versus between genetic variants and exposure) | 14,16 | We visualize results using a forest plot in Fig 2-3 |
| 12 | **Assessment of assumptions** |  |  |  |
|  | a) | Report the assessment of the validity of the assumptions | 8-10 | To assess the validity of the assumptions we took the measures described in point 7; We selected variants that were strongly associated with the exposures at GWAS significance i.e. P< 5×10^-8^ to comply with assumption 1. We additionally used a large number of robust methods such as weighted median, MR-Egger and sensitivity analyses to probe into potential violations of assumption 3, mainly due to horizontal pleiotropy. We also tested for association of IVs with secondary traits and repeated our main analysis without these IVs. Moreover, we carried out a leave-one-out analysis to scrutinize the dependability of our results. In this analysis, we excluded one single nucleotide polymorphism at a time and performed inverse variance weighting analysis on the remaining SNPs to assess their robustness. Our meticulous analysis did not uncover any instrumental variables that deviated significantly from the norm, indicating the reliability of our results.  Detailed information can be found in the Methods section of original paper. |
|  | b) | Report any additional statistics (e.g., assessments of heterogeneity across genetic variants, such as *I^2^*, Q statistic or E-value) | No Applicable | No Applicable |
| 13 | **Sensitivity analyses and additional analyses** |  |  |  |
|  | a) | Report any sensitivity analyses to assess the robustness of the main results to violations of the assumptions | S4-5 Tables | Detailed information can be found in the Results section of original paper. |
|  | b) | Report results from other sensitivity analyses or additional analyses | S6-7 Table | Detailed information can be found in the Results section of original paper. |
|  | c) | Report any assessment of direction of causal relationship (e.g., bidirectional MR) | No Applicable | No Applicable |
|  | d) | When relevant, report and compare with estimates from non-MR analyses | 22-25 | Our genetic discoveries concur with estimates obtained from potential observational investigations that have analyzed the correlation between glucosamine utilization and risk of skin cancer, melanoma, and also type 2 diabetes. In contrast with the findings presented in the preceding observational study, our genetic assessments illustrate that glucosamine intake is positively correlated with a 6% heightened risk of lung cancer (OR 1.06, 95% CI: 0.96-1.17), a decrease of 7% in prostate cancer risk (OR 0.93, 95% CI: 0.85-1.01) and an increase of 1% in COPD risk (OR 1.01, 95% CI: 1.00-1.01).  Detailed information can be found in the Results section of original paper. |
|  | e) | Consider additional plots to visualize results (e.g., leave-one-out analyses) | No Applicable | No Applicable |
|  | **DISCUSSION** |  |  |  |
| 14 | **Key results** | Summarize key results with reference to study objectives | 11-12 | In contrast to a purely protective correlation between glucosamine consumption and risk of cancer and non-neoplastic diseases, our results indicate a deviation from the generally accepted biological understanding, but rather the dichotomous effects of habitual glucosamine ingestion on disease.  Our findings evidence that the intake of glucosamine may pose a risk for several diseases, such as melanoma, follicular lymphoma, autoimmune thyroiditis, autoimmune hyperthyroidism, chronic sinusitis, atopic dermatitis, asthma and bipolar disorder.  Detailed information can be found in the Author summary section of original paper. |
| 15 | **Limitations** | Discuss limitations of the study, taking into account the validity of the IV assumptions, other sources of potential bias, and imprecision. Discuss both direction and magnitude of any potential bias and any efforts to address them | 28-29 | First, this investigation scrutinized the impact of administering exogenous glucosamine, yet it seems improbable that this alone offers a comprehensive explanation for the influence of overall glucosamine levels in the body, encompassing the endogenous aspect. Second, the pool of genetic instruments available for investigating habitual glucosamine consumption is presently restricted, comprising a mere ten genetic variants. This circumstance may have repercussions on the ability to detect pleiotropy through employment of MR Egger methods—although none of our pleiotropy tests disclosed statistically significant infringements, these diagnostic assessments are liable to suffer from insufficient statistical power. Hence, it is necessary to identify additional instrumental variables associated with habitual glucosamine usage......  Detailed information can be found in the Author summary section of original paper. |
| 16 | **Interpretation** |  |  |  |
|  | a) | Meaning: Give a cautious overall interpretation of results in the context of their limitations and in comparison with other studies | 28-29 | In this MR investigation, we comprehensively evaluated the potential correlations between genetically determined glucosamine intake and risk of a diverse spectrum of non-neoplastic as well as cancerous diseases......  Detailed information can be found in the Discussion section of original paper. |
|  | b) | Mechanism: Discuss underlying biological mechanisms that could drive a potential causal relationship between the investigated exposure and the outcome, and whether the gene-environment equivalence assumption is reasonable. Use causal language carefully, clarifying that IV estimates may provide causal effects only under certain assumptions | 25,26 | A wealth of scientific research has explored the anti-cancer mechanisms that reinforce the seemingly indomitable position of regular glucosamine use as a prominent anti-cancer agent......  Detailed information can be found in the Discussion section of original paper. |
|  | c) | Clinical relevance: Discuss whether the results have clinical or public policy relevance, and to what extent they inform effect sizes of possible interventions | 27 | As per the aforementioned premise, the present investigation casts doubt on the purported purely beneficial association between glucosamine ingestion and both neoplastic and non-neoplastic diseases, highlighting instead the potential for unfavorable outcomes......  Detailed information can be found in the Discussion section of original paper. |
| 17 | **Generalizability** | Discuss the generalizability of the study results (a) to other populations, (b) across other exposure periods/timings, and (c) across other levels of exposure | 29.30 | Sixth, although MR analysis can provide insight into the lifetime impact of habitual glucosamine use on cancer and non-neoplastic disorders, the clinical significance of such estimates regarding age-specific interventions is limited. To address this limitation, it would be beneficial to conduct future MR analyses with a gender- or age-specific focus, utilizing larger sample sizes in order to provide more meaningful results.  Detailed information can be found in the Discussion section of original paper. |
|  | **OTHER INFORMATION** |  |  |  |
| 18 | **Funding** | Describe sources of funding and the role of funders in the present study and, if applicable, sources of funding for the databases and original study or studies on which the present study is based | 44 | Detailed information can be found in the Funding section of original paper. |
| 19 | **Data and data sharing** | Provide the data used to perform all analyses or report where and how the data can be accessed, and reference these sources in the article. Provide the statistical code needed to reproduce the results in the article, or report whether the code is publicly accessible and if so, where | 44 | Detailed information can be found in the Data Availability Statement section of original paper. |
| 20 | **Conflicts of Interest** | All authors should declare all potential conflicts of interest | 444 | The authors declare that they have no conflict of interest. |

This checklist is copyrighted by the Equator Network under the Creative Commons Attribution 3.0 Unported (CC BY 3.0) license.

1. Skrivankova VW, Richmond RC, Woolf BAR, Yarmolinsky J, Davies NM, Swanson SA, et al. Strengthening the Reporting of Observational Studies in Epidemiology using Mendelian Randomization (STROBE-MR) Statement. JAMA. 2021;under review.

2. Skrivankova VW, Richmond RC, Woolf BAR, Davies NM, Swanson SA, VanderWeele TJ, et al. Strengthening the Reporting of Observational Studies in Epidemiology using Mendelian Randomisation (STROBE-MR): Explanation and Elaboration. BMJ. 2021;375:n2233.
